# Camel Bite Associated with Depressed Skull Fracture with Rapidly Spreading Subgaleal Cellulitis

**DOI:** 10.1155/2020/8393059

**Published:** 2020-05-23

**Authors:** Shaymaa Al-Umran, Ahmad Abdulfattah, Faisal Alabbas, Hosam Al-Jehani

**Affiliations:** Department of Neurosurgery and Neurocritical Care, King Fahad Hospital of the University Dammam University, Al-Khobar, Saudi Arabia

## Abstract

Camel bite represents a minimal proportion, and most of them are from the Middle East countries. Their infectious potential is poorly understood, and the guidelines for antimicrobial treatment are not well developed. We describe a 40-year-old male, who works as a camel herder and was bitten by a camel while he was tying it down which led to a unilateral depressed skull fracture and multiple bilateral teeth-puncture wounds in the scalp. He arrived to our emergency department 3 hours after injury. All the wounds were dry and the skin around them was healthy looking with no subcutaneous collections. CT scan of the head showed depressed skull fracture on the left temporal region. Within 12 hours, the patient developed spreading cellulitis in the scalp. This necessitated an urgent surgical intervention. The added challenge is the presence of a dural breach. Our patient presented a challenge at several levels. He presented early with clean puncture wounds that were treated according to the most agreed upon guidelines. But our novel finding of rapidly spreading cellulitis requires alteration of recommendation towards more aggressive therapeutic attitude including early surgical intervention, especially for those patients suspected of a dural tear with the depressed skull fracture, even if treated with appropriate antibiotics.

## 1. Introduction

Animal bites are a world wide problem that remained elusive to reliable statistics [1, 2]. Among all the mammalian animal bite injuries reported, camel bite represents a minimal proportion and most of them are from the Middle East countries [3]. Camels are well known for biting their handlers [1, 4]^.^ Although herbivorous, camels have canine-like teeth that can cause deep wound punctures and possibly capable of causing bone fractures [1]. In the Middle East, most of the camel bits occur during rutting season which is between November and March, as the camels may be more aggressive and agitated [3, 4]. Camel bites have been described to cause depressed skull fractures [3]. Their infectious potential is poorly understood, and the guidelines for antimicrobial treatment are not well developed. We describe a patient in whom a camel bite caused a depressed skull fracture, but more importantly, rapidly spreading subgaleal infection in the early phase after the bite.

## 2. Case Report

**Figure 1:**
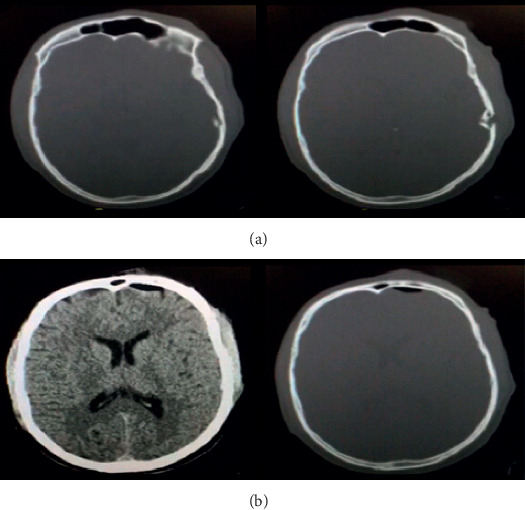
(a) CT of the brain, bone window demonstrating a left temporal depressed fracture with intra-axial bone fragments; (b) CT of the brain demonstrating a left frontal pneumocephalus in both brain window and bone window along with left temporal subgaleal swelling.

The patient is a 40-year-old male, originally from India who works as a camel herder. He reports no chronic medical illnesses especially no diabetes mellitus and no immune suppressive therapy. On the day of admission, he was bitten by an otherwise healthy camel while he was tying it down. He arrived to our emergency department 3 hours after injury. He was found to be vitally stable and afebrile. He was opening eyes spontaneously, obeying command but mildly confused. No lateralizing neurologic deficits were noted on his neurological examination. On scalp inspection, he was found to have 2 small cut wounds on the left side and 3 on the right side both just above the pinna of the ear; all of these wounds are consistent with teeth-bite marks. An abrasion above the left supraorbital region was noted, likely secondary to the fall he sustained after the bite. All the wounds were dry and the skin around them was healthy looking with no subcutaneous collections. He received tetanus toxoid in the ER. Computed tomography scan of the head was obtained and showed depressed skull fracture on the left temporal region associated with pneumocephalus but no intracranial hematoma or contusion were seen (Figure 1). Given his early arrival to our ER, all the wounds were irrigated with normal saline and the patient was started on intravenous flagyl and augmentin immediately to cover oral and skin flora. In addition, phenytoin was started as seizure prophylaxis, given his less-than-perfect initial level of consciousness and the depressed skull fracture over the left temporal lobe. Initial laboratory blood tests were within normal limits. After just 12 hours, the patient was found to be ill-looking with mild fever. The wounds were found to be indurated and with erythematous edges and started to show small amount of lightly purulent discharge. He still maintained his good level of consciousness with a GCS of 15 and showed no focal neurological deficit. He was taken to the operating room for debridement of all wounds. To our surprise, the purulent collection was found to be spreading well beyond the edges of the teeth-puncture wounds into the subgaleal space in the retro-auricular area bilaterally. In the left side, the 2 puncture wounds were connected together to maximize the drainage of the purulent material. The depressed skull fragment was elevated, the dura was sutured after copious irrigation, and the skin was closed primarily. In the right side, the 3 puncture wounds were connected and the pocket of purulent discharge was found to have reached the upper part of the cervical region. As such, the wounds were packed and allowed to heal by secondary intention and were attended to by daily dressing. He continued on antibiotics and wound dressing with close follow-up by our infectious disease colleagues who added rabies vaccine. He was discharged home after 10 days of intravenous antibiotics and was continued on oral antibiotics for another 2 week. He was seen in the outpatient department at that time, and his wounds were healing well with no residual infections.

## 3. Discussion

In most countries, animal bites were reported most frequently by dogs (80%), followed by cats (10%). The rest were reported by many other animal species including the herbivorous camels [4]. Camels are large herbivores with large strong jaws and canine-like teeth [1]. The mechanism of camel bite injury is complex, including penetrating and crushing injuries by the camel teeth and blunt injuries when patients are lifted and thrown to the ground [5]. They usually occur when pulling on the reins of the camels' heads to make them kneel down to allow for mounting. The upper limbs were involved in 64–94% of reported cases [4, 6]; of those, fractures reported to occur in 15–31% of bites while traumatic amputation was seen in 3.3% of bites [4]. Abdominal injuries from camel bites were also described [7].

The rate of infection of bite wounds differs between the animal species due to the difference in the oral flora in the biting animal and the variability of injury type [8]. Animal bite infections should be considered to be polymicrobial, but certain unusual pathogens can be characteristic of particular animal species and knowledge of these is useful to guide antibiotic choice (Table 1). One study was conducted about the prevalence of bacterial species from camel bite where a total of 80 samples were collected from male, female, and young calves of camels and tested for bacterial contamination [6]. Twelve different bacterial species were identified from wound samples of camels [6]. Out of the 80 samples studied, 46 were from males, 12 from females, and 22 from young calves [6]. More than 64 different strains of bacteria were present in the oral cavity of a camel, which could be a potential source of infection [9]^.^ These are reported to be responsible for deep infections such as septic arthritis, osteomyelitis, and tenosynovitis and compartment syndrome [8].

Camel bites associated with depressed skull fractures have been reported in the literature, but there is a lack of consistency as to the need and the timing of the surgical intervention for debridement and skull fracture elevation or dural closure.

As a general recommendation, bite wounds to the face and scalp from any species that are less than 6 hours old may be irrigated and sutured [1]. Puncture wounds, contaminated wounds, wounds more than 12 hours old, or wounds infected at presentation should not be sutured [1]. Patients at risk for infection or poor wound healing are best treated conservatively without suturing [1].

Our patient presented a challenge at several levels. He presented early with clean puncture wounds that were, to our knowledge, treated according to the most agreed upon guidelines. Surprisingly, and only within 12 hours, the patient developed spreading cellulitis in the scalp that spread into the cervical spaces. This necessitated an urgent surgical intervention. The added challenge is the presence of a dural breach in association with the depressed skull fracture. Having not explored the wounds early, the presence of the dural tear would have allowed the spreading of this infection into the intracranial space with a potentially life-threatening meningitis. This was mitigated by the lower threshold for early surgical wound debridement and dural inspection, copious subdural irrigation of the subdural space, and dural closure. Now that the wound is clean, the decision was to follow the guidelines and close the wound. On the contrary, we found expanding and coalescing purulent pockets in addition to the spreading cellulitis all the way to the cervical region in the contralateral side. This could have been overlooked if the 2 sides were thought of as equal picture wounds from the camel teeth. Such a behavior suggests a virulent and aggressive infection that would have most likely failed conservative antimicrobial management.

This case represents the first case in which spreading cellulitis is described along with a depressed skull fracture from a camel bite. The need for vigilant observation and low threshold for surgery is mandatory in such cases to avoid escalation of infection. If more reported cases show the same finding, then one would suggest a change in the recommendation of management of such bites to be inclusive of recommendations towards earlier intervention.

## 4. Conclusion

Camel bites are rarely associated with depressed skull fractures. The finding of the spreading cellulitis is a novel one. In the presence of evidence of dural tear with depressed skull fracture from camel bite, we advocate earlier surgical debridement and elevation of the depressed fragment and repair of the dura, regardless of the condition of the superficial wounds.

## Figures and Tables

**Table 1:** Common organisms and sensitive regimens.

Animal	Organism	Antibiotic	Penicillin allergic	Suturing
Dogs	*Pasteurella dagmatis, P. canis, Staphylococcus aureus, S. intermedius, Streptococci, Moroxella spp, Neisseria spp, Clostridium spp (including Clostridium tetani), Anaerobes spp.*	Augmentin	Moxifloxacin	All (except hands)
		Ceftin	Clindamycin 300 mg PO q6 h × 5 d	

Cats	*Pasteurella multocida, mixed aerobes and anaerobes*	Augmentin	Moxifloxacin	
		Ceftin	Clindamycin 300 mg PO q6 h × 5 d	

Rodents	*Streptobacillus moniliformis, Spirillumminus, Salmonella spp*	No need		Rarely needed

Cows, horses, camels	*Polymicrobial, Actinobacillus spp*	Animal	Animal	Face (as needed)

Monkeys	*Mixed aerobes and anaerobes, Streptococci, Neissria spp, Haemophilus influenzae, Herpes simiae (B virus)*	Keflex 250–500 mg PO q6 h × 5 d	Erythromycin	No need
		Dicloxacillin	TMP-SMX	

Humans	*Viridans streprococci, S.pyogenes, S.aureus, Anaerobes, Eikenella corrodens, hepatitis B and C, HIV*	Augmentin 500 mg PO q12 h × 5 d	Moxifloxacin 400 mg q24 h × 5 d	Face (as needed)
		Ceftin 250–500 mg PO bid × 5 d	TMP-SMX 160 mg PO bid × 5 d	
